# Inhibition of Rotavirus Infectivity by a Neoglycolipid Receptor Mimetic

**DOI:** 10.3390/nu3020228

**Published:** 2011-02-17

**Authors:** Daniel W. Bergner, Theresa B. Kuhlenschmidt, William P. Hanafin, Lawrence D. Firkins, Mark S. Kuhlenschmidt

**Affiliations:** 1 Department of Medicine, Feinberg School of Medicine, Northwestern University, 420 East Superior Street, Chicago, IL 60611, USA; Email: d-bergner@md.northwestern.edu; 2 Department of Pathobiology, College of Veterinary Medicine, University of Illinois, 2001 South Lincoln Avenue, Urbana, IL 61802, USA; Email: tkuhlens@illinois.edu (T.B.K.); firkins@illinois.edu (L.D.F.); 3 Institute for Genomic Biology, University of Illinois, 1206 West Gregory Drive, Urbana, IL 61801, USA; Email: whanafin@illinois.edu

**Keywords:** nutriceutical, carbomimetic, receptor therapy, neoglycolipid, rotavirus, ganglioside, infectious, disease

## Abstract

Group A rotaviruses are a major cause of diarrhea in the young of many mammalian species. In rotavirus infected piglets mortality can be as high as 60%. Previous research in this laboratory has identified a porcine intestinal GM_3_ ganglioside receptor that is required for sialic acid-dependent rotavirus recognition of host cells. In addition, we previously demonstrated exogenously added GM_3_ can competitively inhibit porcine rotavirus binding and infectivity of host cells *in vitro*. Sialyllactose, the carbohydrate moiety of GM_3_, is approximately 3 orders of magnitude less effective than GM_3_ at inhibiting rotavirus binding to cells. Furthermore, production of therapeutic quantities of GM_3_ ganglioside for use as an oral carbomimetic in swine is cost prohibitive. In an effort to circumvent these problems, a sialyllactose-containing neoglycolipid was synthesized and evaluated for its ability to inhibit rotavirus binding and infectivity of host cells. Sialyllactose was coupled to dipalmitoylphosphatidylethanolamine (PE) by reductive amination and the product (SLPE) purified by HPLC. Characterization of the product showed a single primulin (lipid) and resorcinol (sialic acid) positive band by thin layer chromatography and quantification of phosphate and sialic acid yielded a 1:1 molar ratio. Mass spectroscopy confirmed a molecular weight coinciding with SLPE. Concentration-dependent binding of rotavirus to SLPE was demonstrated using a thin-layer overlay assay. Using concentrations comparable to GM_3_, SLPE was also shown to inhibit rotavirus binding to host cells by 80%. Furthermore, SLPE was shown to decrease rotavirus infection of host cells by over 90%. Finally, preliminary results of *in vivo* animal challenge studies using newborn piglets in their natural environment, demonstrated SLPE afforded complete protection from rotavirus disease. The efficacy of SLPE in inhibiting rotavirus binding and infection *in vitro* and *in vivo*, coupled with its relatively low-cost, large-scale production capabilities make SLPE a promising candidate for further exploration as a possible prophylactic or therapeutic nutriceutical for combating rotavirus disease in animals. Most importantly, the results presented here provide proof of concept that the nutriceutical approach of providing natural or synthetic dietary receptor mimetics for protection against gastrointestinal virus infectious disease in all species is plausible.

## 1. Introduction

Rotaviruses are a major cause of diarrhea in the young of most mammalian species and the most important cause of severe dehydrating diarrhea in children worldwide. In excess of 60,000 deaths caused by rotavirus occur in infants and young children annually [1]. Neonatal piglets and calves are very susceptible to naturally occurring rotavirus disease that is often associated with recent weaning. While rotavirus infection is capable of causing significant morbidity and death loss, most infected piglets survive the infection but remain stunted as a result of villous blunting and fusion combined with maldigestion and malabsorption. 

Most herds are infected with porcine rotavirus, especially Group A strains, and there is a disease-producing synergism with intercurrent infection with other enteropathogens. Rotaviruses display a tropism for the villous enterocytes lining the small intestine. Productive infection results in cell lysis. Although some minor variations in the pathophysiological responses exist among species, the most prominent and consistent lesion is villous atrophy. 

Two oral human rotavirus (HRV) vaccines were recently licensed: a three-dose human-bovine reassortant multivalent rotavirus vaccine, RotaTeqTM in the US, Canada and ~30 other countries; and a two-dose attenuated HRV (AttHRV) monovalent vaccine (G1P1A(8)), RotarixTM in more than 90 countries, including countries of the European Union, Latin America, Asia and Africa (for review see [2]). Both vaccines are highly effective against severe rotavirus disease and death in the developed world, and their widespread use has even resulted in reduced disease rates in unvaccinated children [3]. These results are impressive; however, the protective efficacy of these live oral vaccines against cases of rotavirus diarrhea in the developing world is considerably reduced. For example, the approximate protection provided by Rotarix against severe rotavirus diarrhea was 49% in Malawi and 77% in South Africa as compared to 96% in Europe [4,5]. Furthermore, gene reassortment occurs *in vitro* and *in vivo* between human and animal rotavirus [6,7]. With the advent of complete genome sequencing for rotaviruses, there are numerous reports in recent years describing animal/human RV reassortants which have emerged in nature from co-circulating and co-infecting rotaviruses (for review see [8]). Thus, use of HRV vaccines constructed using infectious animal rotaviruses introduces animal rotavirus genes into the human population (for review see [9]). Although it is too early to know whether and to what extent the widespread use of HRV will lead to immune selection of new strains, there is the potential for vaccine-associated collateral infections especially in immunocompromised individuals [10]. In contrast to attenuated-live vaccines, the use of inactivated or non-replicative virus like particles (VLPs) as vaccine candidates, coupled with new strategies for boosting mucosal immunity, [9,11,12,13,14], and/or direct competition with virus-host cell binding using a dietary nutriceutical approach, may have the greatest potential to provide stable, long-term protection against rotavirus disease in both animals and people. This approach also reduces the possibility of emergence of virus P and G types not represented in the vaccine strains since non-replicative virus particles will not reassort with wild type rotaviruses. 

Despite remarkable progress in rotavirus vaccine development for both animals [12,13,15,16,17] and humans [2,18,19,20,21,22], there are no effective commercial vaccines or licensed rotavirus-specific antiviral agents for animals in wide clinical use and no practical method of preventing rotavirus infection in swine herds. In this report, we provide proof of concept that an orally administered, synthetic, neoglycolipid can be used as a therapeutic receptor mimetic for the prevention of Group A rotavirus disease in neonatal piglets. 

## 2. Experimental Section

### 2.1. Cells and Virus

For all *in vitro* experiments, Group A porcine rotavirus (OSU strain (P9(7)G5)) was propagated in MA104 cells (ATCC HTB 37) and triple and double-layered virus particles isolated by gradient purification using the following modification of standard techniques [23,24,25]. A single gradient centrifugation step was performed using a near vertical tube rotor (Beckman, NVT65) for 6.5 h, at 60,000 rpm (291,110× g) instead of dual gradient runs using an SW 55 swinging bucket rotor, at 35,000 rpm, (116,140× g), for 30 h. For *in vivo* studies, the above virus was passed in newborn piglets and partially purified from feces as previously described [26]. 

### 2.2. Synthesis of Neoglycolipids

Sialyllactose or lactose was linked to dipalmitoylphosphatidylethanolamine (PE) to yield sialyllactosylphosphatidylethanolamine (SLPE) or lactosylphosphatidylethanolamine (LPE) via reductive amination using modifications of a previously described procedure [27]. Briefly, 100 mg of sialyllactose (SL) or lactose was dissolved in DMSO (1 mL) and then mixed with 200 mg PE in 40 mL CHCL_3_:MeOH (2:1) under constant stirring in a round bottomed flask. The tube containing the SL was rinsed with methanol (5 mL) and added to the flask, and the reaction mixture was incubated at 60 °C for two hours. At the end of this incubation, 1 mL of reducing agent NaCNBH_4_, (10 mg) dissolved CHCl_3_:MeOH:acetic acid (2:1:0.001, v/v) was prepared fresh and added to the reaction mixture. Four more 1 mL aliquots of reducing agent were added to the reaction at approximately 4 h intervals and appearance of reaction products monitored using analytical thin layer chromatography (TLC) and orcinol, resorcinol, and primulin sprays to identify bands containing neutral carbohydrate, sialic acid, and lipid, respectively. Following approximately 22 h total reaction time, the mixture was dried by rotary evaporation, dissolved in 20 mL water, and dialyzed against 5 L of H_2_O for 5 h. The dialysis was repeated twice, the sample lyophilized, and the SLPE (or LPE) resuspended in 25 mL CHCl_3_:MeOH:H_2_O (65:25:3, v/v) and purified using preparative HPLC (below). 

### 2.3. Purification of Neoglycolipids by Preparative HPLC

Aliquots (5 mL) of SLPE or LPE were filtered 0.45 µm nylon filters and applied to 10 µm silica preparative HPLC column (250 mm × 22 mm, Econosil, Alltech Associates, Inc., cat. #6258). The sample was applied to the column in CHCl_3_:MeOH:H_2_O (65:25:3, v/v) at a flow rate of 2 mL/min and eluted using the following sequence: 0–30 min, 100% CHCl_3_:MeOH:H_2_O (65:25:3, v/v); 30–75 min, a linear gradient of CHCl_3_:MeOH:H_2_O from (65:25:3, v/v) to (55:45:10); 75–120 min, 100% CHCl_3_:MeOH:H_2_O (55:45:10); 120–125 min, a linear gradient of CHCl_3_:MeOH:H_2_O from (55:45:10) to (30:60:20); and from 125 to 160 min, 100% CHCl_3_:MeOH:H_2_O (30:60:20). Fractions were collected every 2 min and continuously monitored by stream splitting and refractive index measurements using an evaporative laser light scattering detector (Varex ELSD MK III, Alltech Associates, Inc.). Detector settings were as follows: drift tube temperature = 60 °C, exhaust temperature = 35.9 °C, gas (N_2_) flow rate = 0.7–0.9 SLPM and pressure = 6.8 psig; the solvent pressure was 0.2 psig. Purity of the HPLC fractions and final SLPE or LPE pool was assessed by analytical TLC [23,24,25]. 

### 2.4. Neoglycolipid Product Characterization

For characterization of the purified SLPE or LPE reaction product, molecular mass and phosphate to sialic acid molar ratios were determined using the following assays. Sialic acid was determined by modification of an HPLC/thiobarbituric acid method [28]. Briefly, following acid hydrolysis and development of the chromagen as originally described, 100 µL aliquots were injected and chromatographed through a 4.6 mm × 250 mm C-18 reverse phase column (Alltech Associates, Inc.). The solvent (2× buffer stock:MeOH:water, 5:3:2, v/v) was run isocratically using a Dionex DX300 HPLC apparatus set at a flow rate of 0.7 mL/min and 549 nm for measuring absorbance of chromagen. Phosphate content was measured colorimetrically [29] and molecular mass was determined using low-resolution fast-atom bombardment mass spectrometry (FAB-MS) at the Mass Spectrometry Laboratory, School of Chemical Sciences, University of Illinois as previously described [30]. 

### 2.5. Synthesis of Sialyllactosyl-BSA

Sialyllactose was coupled to BSA via reductive amination as previously described [31]. The Reaction product was extensively dialyzed *versus* water and then lyophilized. The average number of sialyllactose residues coupled to BSA was 37 and was determined following acid hydrolysis by HPAEC-PAD monosaccharide analysis [25] and protein determination by bicinchoninic acid assay [32]. 

### 2.6. Acetylation of PE and PE Derivatives

Acetic anhydride was added in five portions to samples containing PE, LPE, and SLPE in a 1:1 chloroform:methanol solution containing sodium bicarbonate as previously described [33]. Tubes were incubated at room temperature and the reaction was stopped by the addition of water. The reaction mixture was then extracted 3 times using CHCl_3_:MeOH:H_2_O at a final ratio of 1:1:1.1 (v/v), the organic layers pooled and evaporated to dryness. Reaction products were characterized using analytical TLC [24,25]. 

### 2.7. Virus Radiolabeling and Host Cell Binding Assay

Virus (TLP) radiolabeling with ^125^I was performed as previously described [23,25]. Viral protein was quantified colorimetrically [32] with human serum albumin as the standard (Micro BCA; Pierce Chemical, Co.). Measurement of virus binding to MA104 cells in the absence or presence of added gangliosides or other glycoconjugate inhibitors was performed based on the results of binding kinetic and virus dose experiments as previously described [25]. VP6 was released from porcine double-layered rotavirus particles (DLP) after treatment with 1 M CaCl_2_ as previously described [34]. Briefly, 400 µg DLP from porcine OSU strain in 3 mL TNC buffer (50 mM Tris, 150 mM NaCl, 10 mM CaCl_2_, pH 7.5) were sonicated as described. The sample was made 1 M in CaCl_2_ by addition of 440 mg CaCl_2_ and was incubated at 37 °C for 20 min. After sonication, the sample was centrifuged at 192,000× g for 1 h at 4 °C to separate the core particle (pellet) from released VP6 proteins (supernatant). The supernatant was dialyzed against distilled water at 4 °C. After concentration by evaporation with a gentle stream of air, protein concentration was determined and 10 µg was radioiodinated as described above. SDS PAGE and autoradiographic visualization of separated radioactive proteins was performed using standard techniques as previously described [35]. 

### 2.8. Virus-Glycolipid Binding Assay

Measurement of ^125^I-labeled rotavirus (TLP) binding to immobilized gangliosides (0.5–10 nmol) or neoglycolipids (SLPE, LPE, or acetylated derivatives (~750 pmol)) was measured using a thin-layer plate overlay assay as previously described [25]. Briefly, glycolipids were applied to the origin of a plastic-backed silica gel TLC plate which had been pre-run in chloroform-methanol (MeOH)-H_2_O (60:30:4.5, v/v). The plate was developed in chloroform-MeOH-H_2_O (55:45:10), and after it was air dried, overlay of the ^125^I-labeled rotavirus was performed by a modification of a previous method [36]. The developed plate was treated with ice-cold TNC-BSA buffer (TNC plus 1% bovine serum albumin (Sigma; catalog No. 7030)) for 1 h at 4 °C. The plate was then rinsed quickly in TNC buffer, placed in a glass dish with 7 mL of TNC containing 1.25 × 10^7^dpm (~3 µg of viral protein) of ^125^I-labeled rotavirus (TLP)/100-cm^2^ plate, and gently rocked for 2.5 h at 4 °C. The ^125^I-rotavirus overlay was removed by aspiration, and the plate was rinsed seven times in 400 mL of ice-cold filtered TNC. After air drying for 1 h at room temperature, the plate was wrapped in plastic, placed in a Cronex Lightning Plus cassette with intensifier screens, exposed to Kodak X-Omat film at −80°C for 4 h, and developed in an automatic developer. 

### 2.9. Measurement of *in Vitro* Virus Infectivity: Focus-Forming Assay

Virus focus forming unit (FFU) assays were performed using confluent monolayers of MA104 cells in 24 well plates as previously described [25]. Briefly, gradient purified triple-layered virus particles were treated with trypsin (Sigma T0134, porcine pancreas at a final concentration of 10 µg/mL (152 BAEE units/mL)) for 30 min at 37 °C. Dilution of the trypsinized virus was performed to yield approximately 500 FFU/100 µL. Aliquots of diluted virus were incubated with either PE, glyco-PE derivatives, gangliosides, or were sham-treated for 15 min on ice. Treated virus suspensions (100 µL) were inoculated into wells followed by incubation at 37 °C for 30 min. One milliliter of ice cold MEM was then added, aspirated and replaced with 1 mL of fresh MEM. The plates were warmed to 37 °C and incubated as described above for 16–18 h. Virus infectivity was quantified using rotavirus-specific, immunoperoxidase histochemical staining and counting of stained foci under light microscopy.

### 2.10. Animal Infectivity Experiments

Term pregnant normal, rotavirus antibody negative sows (University of Illinois Veterinary High Health, Swine Herd) were induced to labor approximately 18 h prior to farrowing. Before the newborn piglets were allowed to nurse, one group from the litter was inoculated with rotavirus doses ranging from 1 × 10^3^ to 1 × 10^5^ focus forming units (FFU)/mL MEM *per os*. Another set of piglets from the same litter was orally inoculated with the identical virus doses as the first set but including 1–2 µmol SLPE/mL MEM (in separate experiments) either as a single dose or by continuous feeding (every 12 h). All piglets were dosed within 2 h of birth. The piglets were then returned to their respective sow and littermates housed in separate crates (for SLPE *versus* control (MEM) fed groups), but in the same farrowing room, for the remainder of the experiment. This experimental design was used in an effort to mimic the environment found in commercial pig production facilities. Fecal material from each piglet was collected by anal swab 24–96 h after initial virus inoculation and enzyme linked inmunoassays (ELISA), for detection of rotavirus antigen (group A-specific), were performed on the collected fecal material using an IDEIA kit (cat. #K6020, DakoCytomation) according to manufacturer’s instructions. In some experiments, piglets were euthanized at various times postinoculation, intestinal contents collected, and sections of small intestine were processed for immunohistochemistry to localize rotavirus antigen as previously described [37]. Sows were sold and not returned to the herd at the conclusion of the experiments. All animal procedures described in this study were performed in accordance with protocols approved by the University of Illinois Institutional Animal Care and Use Committee (IACUC).

## 3. Results and Discussion

### 3.1. Synthesis, Purification, and Characterization of Sialyllactosylphoshatidylethanolamine

**Figure 1:**
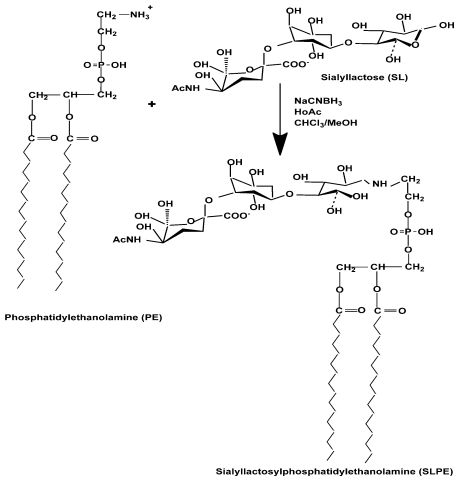
Scheme for synthesis of SLPE. SLPE is synthesized using a one step reductive amination to covalently couple the amino group of phosphatidylethanolamine to the anomeric carbon of the reducing end glucose residue of sialyllactose.

Our earlier results [25] demonstrated that GM_3_, particularly *N*-glycolylGM_3_, ganglioside serves as a primary intestinal receptor for porcine Group A (OSU) rotavirus. In addition, we demonstrated the binding affinity for free sialyllactose, the virus binding epitope of GM_3_, was three orders of magnitude lower than for the intact ganglioside [35]. Based on these results and the desire to produce an efficacious receptor mimetic to protect against rotavirus disease, we set out to synthesize a neoglycolipid, receptor mimetic that could mimic the activity of the natural ganglioside receptor and be produced in large enough quantities for use as a possible nutriceutical. In devising the synthetic scheme for this neoglycolipid (Figure 1) we sought to reproduce the amphipathic character of the natural GM_3_, ganglioside receptor, by substituting the sphingosine lipid moiety of GM_3_, which is not directly involved in virus binding, with a readily-available lipid such as phosphatidylethanolamine that contains a reactive functional group, e.g., an amino group. We previously hypothesized the function of the lipid moiety of GM_3_, relative to its ability to inhibit rotavirus infectivity of host cells, is to enable presentation of the sialyllactose virus-binding epitope in multivalent form in aqueous media. This multivalent presentation is likely responsible for the observed increased virus binding avidity of GM_3_ compared to free sialyllactose [25]. Accordingly, synthetic sialyllactosylphosphatidylethanolamine (SLPE) was designed to contain the sialyllactose virus-binding epitope coupled to phosphatidylethanolamine in an effort to mimic the amphipathic nature of the native ganglioside receptor. For this purpose, a one-step reductive amination synthesis scheme was used as previously described [27,38]. 

**Figure 2:**
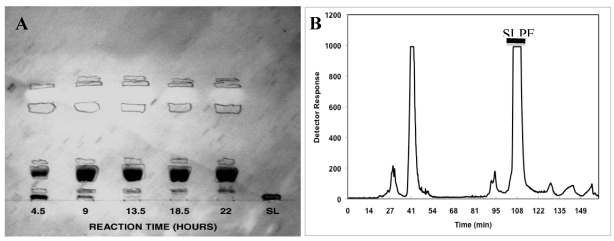
SLPE synthesis time course and purification. (**A**) aliquots of the reductive amination reaction were spotted on TLC plates and stained with primulin followed by resorcinol to detect lipids and sialic acid, respectively. Dark bands are resorcinol positive while circled areas correspond to primulin staining bands; (**B**) purification of SLPE by preparative HPLC was performed as described in the Experimental Section. Fractions from 104–112 min were pooled and analyzed for SLPE purity by analytical TLC (Figure 3).

**Figure 3:**
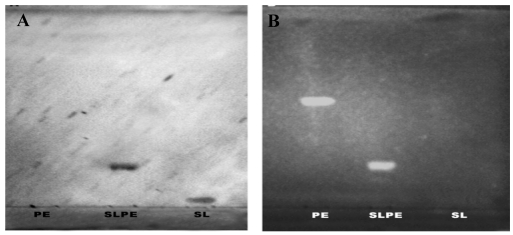
Evaluation of the purity of SLPE. The purity of SLPE following synthesis and HPLC purification was assessed by analytical TLC as described in the Experimental Section. (**A**) TLC chromatogram stained by resorcinol spray to detect sialic acid-containing bands. The chromatographic mobility of SLPE is shown along with standard PE and SL. (**B**) a parallel chromatogram as described in A was run, stained with primulin, and photographed under UV light to detect lipid-containing bands.

Characterization of the reductive amination product by thin-layer chromatography (TLC) of aliquots, taken at various times during the reaction, show clear development of a major primulin (lipid) and resorcinol (sialic acid) staining band (Figure 2). Also, complete disappearance of the initial sialyllactose band in the reaction mixture is evident after 13.5 h incubation. Following purification of the putative SLPE product by preparative HPLC (Figure 2), pooled HPLC fractions show a single primulin and resorcinol positive band with no traces of PE or sialyllactose present (Figure 3). The molar ratio of phosphate to sialic acid in this product was 1:1. Similar results were obtained for the synthesis of LPE using the same reductive amination conditions but substituting lactose for sialyllactose. Characterization of this product using orcinol (neutral sugar) and primulin staining for TLC as well as quantification of the molar ratio of galactose and phosphate in the purified HPLC fraction (1:1 galactose:phosphate, data not shown) indicated the expected product was obtained.

**Figure 4:**
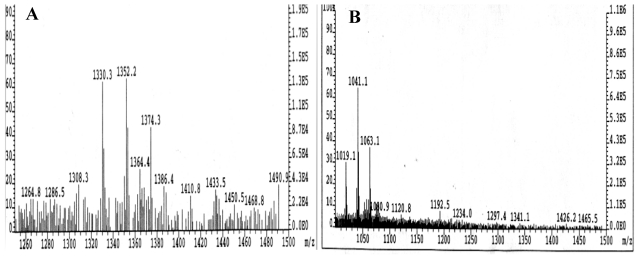
Characterization of SLPE and LPE by FAB mass spectrometry. Low resolution FAB mass spectrometry was performed on TLC/HPLC-purified SLPE and LPE as described in the Experimental Section. (**A**) SLPE; (**B**) LPE.

Finally, low resolution FAB mass spectroscopy of the purified SLPE fraction yielded a parent ion of 1308.3 with corresponding sodium salt peaks of 1330.3, 1352.3, and 1374.3 (Figure 4A). This parent ion molecular mass corresponds to the molecular mass of the proposed sialyllactosyl-dipalmitoylphosphatidylethanolamine product. Similar results were obtained for the purified LPE reaction product (Figure 4B) and a parent ion mass of 1019.1 with sodium salt peaks of 1041.1 and 1063. These values are in agreement with the predicted molecular mass of lactosyldipalmitoylphosphatidylethanolamine. 

### 3.2. Rotavirus Binding to SLPE and LPE Neoglycolipids

**Figure 5:**
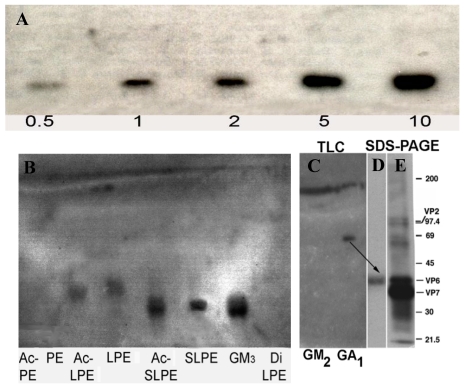
Binding of rotavirus to SLPE and LPE and their acetylated derivatives. Binding of rotavirus (TLP) to the indicated neoglycolipids and their acetylated derivatives was performed by TLC overlay and SDS PAGE as described in the Experimental Section. Panel **A** shows rotavirus binding to increasing concentrations (0.5–10 nmol) of SLPE. Panel **B** compares the relative binding affinity of [^125^I]rotavirus TLP to SLPE, LPE and their acetylated derivatives. Panel **C** depicts the binding of purified [^125^I]VP6 protein to GM_2_ and asialo-GM_1_ ganglioside (GA_1_). Panel **D** shows the electrophoretic mobility and autoradiography of radioactive material recovered from the band (arrow) scraped from the GA_1_ lane of the TLC plate in Panel C. Panel **E** shows the separation and visualization of [^125^I]rotavirus TLP proteins by SDS PAGE and autoradiography. Note the GA_1_ binding radioactivity recovered from Panel D co-migrates with rotavirus VP6 capsid protein.

To determine if rotavirus was able to recognize either SLPE or LPE we used TLC overlay binding assay as described in the Experimental Section. Rotavirus bound to SLPE in a concentration-dependent manner (Figure 5A). Interestingly, rotavirus appeared to bind with slightly greater affinity to the acetylated derivative of SLPE. This may be due to the removal of the amino group positive charge by *N*-acetylation. Although, the impact of *N*-acetylation on SLPE function as a receptor mimetic was not further explored in this study, it may be worthwhile in future studies to examine additional SLPE derivatives in attempts to further enhance rotavirus binding affinity. Rotavirus also bound to both LPE and acetyl-LPE, although this binding appeared weaker than to SLPE. The binding to LPE derivatives is likely due to the asialoganglioside specific binding activity of rotavirus double layered particles [39] which we show is attributable, at least in part, to inner capsid protein VP6 (Figure 5B). No rotavirus binding was observed for PE, acetyl-PE, or Di-lactosylPE (DiLPE) (Figure 5B). 

### 3.3. SLPE Inhibits both Rotavirus Binding and Infectivity of Host Cells *in Vitro*

**Figure 6:**
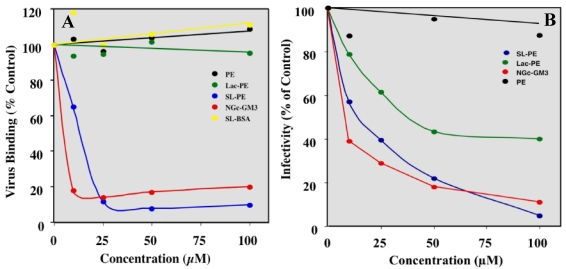
Effect of SLPE and LPE on rotavirus binding and infectivity of MA104 cells. (**A**) Virus (RV TLP) binding and (**B**) infectivity of MA104 cells in the presence and absence of various concentrations of SLPE, LPE, and other lipids or neoglycoconjugates were measured as described in the Experimental Section. (●), PE; (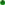), LPE; (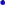), SLPE; (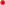), NGcGM_3_; (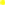), Sialyllactose-BSA (SL-BSA). SL-BSA concentrations are based on µM equivalent of sialic acid.

SLPE displayed dose-dependent inhibition of both rotavirus binding and infectivity of MA104 cells that was comparable to native GM_3_ ganglioside (Figure 6). SLPE inhibited approximately 90% of virus binding and infectivity of MA104 cells at concentrations of 25 and 80 µM, respectively. The inhibitory concentration for 50% inhibition of virus infectivity (IC_50_) was approximately 15 µM for SLPE as compared to 5 µM for GM_3_. No inhibition of virus binding was observed for LPE, the neoglycoprotein sialyllactoseBSA, or PE. 

These results indicate the most potent inhibitors or rotavirus binding are glycolipids rather than glycoproteins even though the sialyllactoseBSA is multisubstitued with sialic acid epitopes. Somewhat surprisingly, LPE was shown to inhibit 50% of virus infectivity at concentrations 50 µM or above. Similarly, acetyl-LPE also inhibited infectivity to a similar extent (data not shown). PE showed no significant inhibition of virus infectivity (Figure 6). The mechanism by which LPE inhibits rotavirus infectivity is unknown but intriguing given the fact it displays only weak binding to the virus in TLC overlay assays and no inhibition of virus-host cell binding (Figure 6). Others and we previously demonstrated purified, non-infectious rotavirus double-layered particles (DLP) bind asialogangliosides [35,39]. Here we demonstrate purified, radioiodinated VP6 binds asialoganglioside GA_1_ (Figure 5C). These results suggest the weak binding of rotavirus TLP to LPE observed in TLC overlay assays (Figure 5B), may involve VP6 binding to non-reducing galactose residues on LPE. Thus, it is possible LPE-mediated inhibition of virus infectivity may interfere with either virus entry or replication rather than initial, sialic acid-dependent binding and is an especially interesting hypothesis that may explain why LPE is able to partially block rotavirus infectivity without inhibiting host cell binding (Figure 6). 

### 3.4. SLPE Inhibits Rotavirus Infection and Disease in Piglets

For experiments aimed at testing the ability of the SLPE to block rotavirus infection *in vivo*, relative large amounts (~90 mg) of SLPE were produced to test as a nutriceutical for protection against rotavirus disease at the time of virus infection in neonatal piglets. In the first experiment, each piglet received either 1.0 µmol SLPE in MEM, or MEM alone, as a single dose *per os* at the time of virus inoculation. The presence of clinical signs was monitored for 88 h post-inoculation (PI) and virus shedding was measured at 40 h, 64 h, and 88 h as described in the Experimental Section. In the second and third experiment, each piglet was dosed with SLPE or MEM at the time of virus inoculation as described above and then again every 12 h for up to 96 h at which time all piglets were euthanized. Clinical signs, virus shedding and immunohistochemical detection of rotavirus in intestinal sections harvested from euthanized piglets were analyzed as described in the Experimental Section. The comparison of the amount of rotavirus shed in feces of experimental (+1.0 µmol SLPE) and control piglets (MEM only), at 40 h, 64 h, and 88 h post infection with varying amount of virus (1 × 10^3^–1 × 10^5^ FFU), is shown in Table 1. 

**Table 1:** Effect of single dose SLPE on virus shedding in rotavirus inoculated piglets.

Piglets	Virus dose (FFU)	SLPE (µmol)	ELISA 40 h PI (% control)	ELISA 64 h PI	ELISA 88 h PI	Diarrhea
**1**	1 × 10^5^	-	2.704 (100)	0.938	1.449	+
**2**	1 × 10^5^	1.0	0.931 (34)	0.298	0	−
**3**	1 × 10^4^	-	2.684 (100)	1.026	0.218	+
**4**	1 × 10^4^	1.0	0.845 (31)	0	1.683	−, + (88 h)
**5**	1 × 10^3^	-	2.520 (100)	0	0.009	+
**6**	1 × 10^3^	1.0	0.390 (15)	0	1.358	−, + (88 h)

This first experiment was designed to determine optimal virus dose and initially evaluate whether SLPE afforded any protection against virus shedding and diarrhea. SLPE-treated piglets did not develop clinical signs of rotavirus disease (diarrhea, lethargic or huddling behavior, anorexia) compared to their littermates receiving only MEM, who developed watery diarrhea, fecal staining, huddling behavior, and anorexia beginning 24 h post-inoculation. Although virus shedding was observed in piglets receiving a single dose of SLPE it was markedly (66–85%, depending on virus dose) reduced in SLPE fed piglets 40 h PI (Table 1). Virus shedding continued at 64 h post inoculation in the SLPE fed pig that received the highest dose of virus and the two SLPE-fed piglets receiving the lower virus doses shed virus antigen again at 88 h post inoculation (Table 1). Although the number of animals in this initial experiment was small, comparison of the ELISA results at 40 h, 64 h and 88 h PI suggested the protection from initial rotavirus infection (or re-infection from littermates, see discussion below) and virus shedding afforded by a single dose of SLPE began to wane sometime after 40 h PI with the highest virus dose (1 × 10^5^ FFU). 

In a subsequent experiment, 11 control piglets (inoculated with 1 × 10^5^ FFU virus alone) and 9 experimental piglets (inoculated with 1 × 10^5^ FFU plus a single dose of SLPE (1 µmol)) were maintained on separate sows but in the same farrowing house. All control piglets displayed a positive fecal ELISA (average value = 0.727, range = 0.100–2.50) compared to none of the SLPE-treated piglets (average ELISA value = 0.001, range = 0.000–0.007) at 24 h post inoculation. At 48 h post inoculation; however, 6/9 SLPE-fed piglets had positive fecal ELISA values. Together, the results of these two experiments indicated a single dose of SLPE provided some initial protection against rotavirus infection, as evidenced by the delay in virus shedding in feces, but was not able to provide sustained protection. 

**Figure 7:**
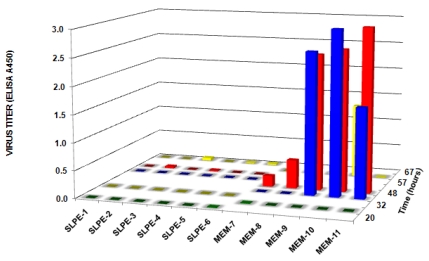
Effect of continuous feeding SLPE on virus shedding in rotavirus inoculated piglets. Piglets were inoculated with rotavirus (1 × 10^5^ FFU) in the presence or absence of SLPE (1 µmol) fed every 12 h, and virus shedding in feces determined as described in the Experimental Section.

Based on the above results, we tested whether continuous feeding SLPE (feeding every 12 h) would afford greater protection against rotavirus infection and disease. When SLPE (1 µmol) was fed every 12 h post-inoculation for the duration of the experiment, virus shedding in feces was not detected in any of the experimental piglets (Figure 7). In contrast, all control piglets challenged with virus in the absence of SLPE feeding shed virus between 32 and 67 h and displayed clinical signs of rotavirus disease (diarrhea). The time of onset of fecal shedding and diarrhea in the control piglets (rotavirus only inoculated) was quite variable and may be explained by differences in colostrum intake or virus dose resulting from re-exposure through contact with diarrheic and virus shedding littermates.

**Figure 8:**
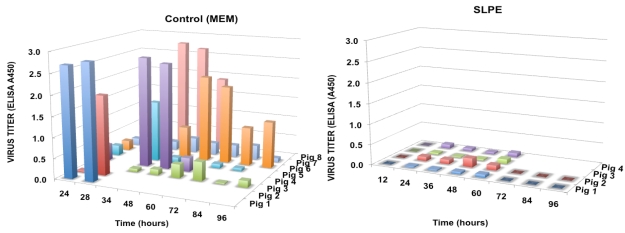
Effect of feeding SLPE (2 µmol) every 12 h on virus shedding in rotavirus inoculated piglets. Piglets were inoculated with rotavirus (1 × 10^5^ FFU) in the presence or absence of SLPE fed every 12 h, and virus titers in feces collected at the indicated times determined as described in the Experimental Section.

In the fourth experiment, SLPE (2 µmol) was continuously fed (every 12 h) for 96 h as described above. Again, rotavirus disease and virus shedding were not detected in piglets fed SLPE as compared to control piglets inoculated with virus alone (Figure 8). In the control piglets, the onset of rotavirus disease and virus shedding was again variable but all of these piglets shed virus at some point during the 96-h experiment (Figure 8). As was the case in the previous experiment (Figure 7), the onset of virus shedding was variable and is probably explained by different amounts of colostrum intake, or re-infection via transmission among littermates following initial virus inoculation. Such a scenario is likely since all piglets were returned to their respective sows following initial virus inoculation and after each feeding. Although both sows and litters (SLPE-fed and virus alone) were housed in separate crates, they were in the same farrowing room and shared a common air supply. Accordingly, these results are encouraging considering none of the SLPE-fed piglets developed disease or shed virus despite the potential of being exposed to rotavirus from the neighboring virus-shedding control piglets.

**Figure 9:**
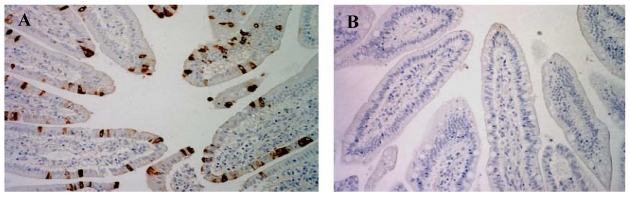
.Effect of SLPE feeding on immunohistochemical detection of rotavirus in intestinal tissue from rotavirus-infected piglets. Piglets were inoculated with rotavirus (1 × 10^5^ FFU) in the presence of MEM or SLPE (2 µmol) and fed MEM or SLPE (2 µmol) every 12 h, euthanized 28–96 h post-inoculation, and intestinal tissue harvested and processed for immunohistochemical detection of rotavirus antigen as described in the Experimental Section. Typical photomicrographs of immunohistochemically stained sections of small intestinal segments from control and SLPE-fed, virus-infected piglets are shown below. (**A**) rotavirus-inoculated pig fed MEM (40 h); (**B**) rotavirus-inoculated pig fed SLPE (40 h).

Finally, rotavirus infection was clearly evident following immunohistochemical staining of intestinal tissue harvested from control virus-infected piglets, but not in virus-inoculated piglets continuously fed SLPE (Figure 9). In piglets infected with rotavirus but in the presence of 1–2 µmol of SLPE and then repeatedly fed SLPE every 12 h, almost no infected enterocytes were detected in intestinal tissue. Although we have not conducted separate experiments designed to evaluate the safety of continuously feeding SLPE to newborn piglets, we found no evidence of any toxicity associated with SLPE feeding in virus-inoculated piglets prior to virus shedding or development of diarrhea. SLPE piglets behaved normally and continued to gain weight (0.058 kg/day) comparable to piglets not receiving SLPE (0.048 kg/day). Clearly, studies aimed at evaluation the safety of SLPE feeding need to be conducted prior to performing large scale clinical or field efficacy trials.

## 4. Conclusions

Our findings demonstrate a dramatic reduction in virus shedding and absence of rotavirus disease in SLPE-fed piglets, thus providing “proof of concept” that a nutriceutical approach of using natural or synthetic receptor mimetics, such as SLPE, hold substantial promise for the prevention of rotavirus disease. This approach may be applicable not only to piglets in a natural field setting, but also as a potential dietary supplement, for example in infant formulae, for the prevention of rotavirus or other gastrointestinal infectious diseases in human infants. 
